# Immediate Postoperative Weightbearing Following Arthroscopic Bone Marrow Stimulation for Talar Osteochondral Lesions: A Matched Cohort Study

**DOI:** 10.1177/10711007251348196

**Published:** 2025-07-10

**Authors:** Tristan M. F. Buck, Jari Dahmen, Quinten G. H. Rikken, Julian J. Hollander, Sjoerd A. S. Stufkens, Gino M. M. J. Kerkhoffs

**Affiliations:** 1 Amsterdam UMC location University of Amsterdam, Departement of Orthopedic Surgery and Sports Medicine, Amsterdam, the Netherlands; 2 Amsterdam Movement Sciences, Musculoskeletal Health, Amsterdam, the Netherlands; 3 Academic Center for Evidence-based Sports medicine (ACES), Amsterdam, the Netherlands; 4 Amsterdam Collaboration on Health & Safety in Sports (ACHSS), IOC Research Center, Amsterdam, the Netherlands

**Keywords:** osteochondral lesion of the talus, OLT, ankle, cartilage, rehabilitation

## Abstract

**Background:**

Bone marrow stimulation (BMS) is the most frequently performed surgical procedure for osteochondral lesions of the talus (OLTs). After the surgical intervention, one of the first goals of rehabilitation is to resume weightbearing. This study aims to compare clinical and radiologic outcomes between immediate weightbearing and delayed weightbearing, which represent unrestricted weightbearing and weightbearing starting at 6 weeks postoperatively.

**Methods:**

All patients who underwent BMS for their OLT between July 2019 and September 2022 in our clinic were screened for eligibility. Patients were retrospectively included with prospective collected data and were matched into 2 groups, the immediate weightbearing group or the delayed weightbearing group. The following variables were used for matching: age, gender, side, lesion size (volume and surface measured on CT scans), primary or nonprimary lesion, body mass index (BMI) and the numeric rating scale (NRS) of pain during walking. The primary outcome of this study is the comparison of the change in NRS of pain during walking between baseline and 12 months postoperatively, between both groups. Secondary outcomes consist of change in the NRS of pain during running, NRS pain during stairclimbing, NRS pain during rest, 36-Item Short Form Health Survey, Foot and Ankle Outcome Score, return to work, return to sport, and radiologic outcomes between both groups at 12 months.

**Results:**

After matching, 13 patients per group were included. Both groups showed improvement in NRS pain during walking from baseline to 12 months postoperatively. The difference in change scores between immediate and delayed weightbearing was not statistically significant (*P* = .57, 95% CI −3.25 to 1.86). A higher proportion of patients in the immediate weightbearing group exceeded the minimal clinically important difference threshold of 2 points compared with the delayed group (OR = 1.9, 95% CI 0.30-11.7), although this was not statistically significant. No significant between-group differences were observed in secondary clinical or radiologic outcomes, nor in return-to-work or return-to-sport rates.

**Conclusion:**

This matched cohort study found no statistically significant difference in clinical or radiologic outcomes at 12 months between immediate and delayed weightbearing following arthroscopic BMS for talar osteochondral lesions. Although early weightbearing may be feasible and well tolerated, the small sample size and wide CIs limit the strength of conclusions. These findings should be considered hypothesis-generating and underscore the need for larger, prospective trials.

**Level of Evidence:** Level III, retrospective matched cohort study.

## Introduction

Bone marrow stimulation (BMS) is the most frequently performed surgical procedure for osteochondral lesions of the talus (OLTs).^4^ This procedure aims to alleviate symptoms by facilitating sufficient bony filling and the formation of fibrocartilage following debridement of an OLT. After surgical intervention, rehabilitation is required to regain ankle function.^1^ One of the first steps in rehabilitation is resumption of weightbearing. As it is known that loading plays a role in the quality and volume of osteochondral lesion repair tissue, postoperative weightbearing after BMS is an important topic.^2^ Historical assumptions that a certain period of nonweightbearing (NWB) is required to facilitate cartilage restoration after BMS has led to a more conservative management in the postoperative treatment strategies after BMS.^13^

In the current literature, resumption of weightbearing is divided into 2 different strategies: early weightbearing and delayed weightbearing. Different definitions exist for early weightbearing and delayed weightbearing.^20^ Early weightbearing is considered as resumption of (partial) weightbearing between day 1 and week 2 postoperative, whereas delayed weightbearing is considered as resuming (partial) weightbearing from week 6 or later.^20^ In the current literature, most articles support the benefits of resumption of partial weightbearing within the first week and resuming full weightbearing at 6 weeks.^6,8,11^

Both early weightbearing and delayed weightbearing yield satisfactory clinical outcomes (ie, pain and function) and no differences between these 2 strategies were observed in prior studies.^5,11^ The clinical advantage of early weightbearing relies on the fact that patients are less restricted in their activities, which possibly leads to higher satisfaction rates and improved (early) clinical outcomes. Another advantage of early weightbearing is that movement restrictions can be avoided as patients with weightbearing constraints often demonstrate noncompliance in relation to weightbearing restrictions.^3,15^ Additionally, it can lead to earlier return to sport and return to work compared with delayed weightbearing. However, the literature on early weightbearing is scarce. To our knowledge, no prospective registered comparative studies comparing early weightbearing, considered as immediate unrestricted weightbearing, with delayed weightbearing (6 weeks nonweightbearing) without bracing or splinting have been published. Additionally, outcomes on return to work and return to sport are limited. The aim of this study is to add knowledge on clinical outcomes and safety of immediate weightbearing after arthroscopic BMS.

## Methods

This study is a retrospective analysis of prospectively collected data. The study was approved by the Medical Ethics Committee at the University of Amsterdam (reference number: 08/326) and is written in accordance with the Declaration of Helsinki. Informed consent was obtained from all patients included in the study.

### Patient Selection

All patients who underwent arthroscopic BMS for OLTs in our clinic between July 2019 and September 2022 were screened for eligibility. The inclusion and exclusion criteria are presented in Table 1. After applying the inclusion and exclusion criteria, patients were matched into 2 groups, the immediate weightbearing group or the delayed weightbearing group, based on received postoperative weightbearing protocol. Both protocols are described in Table 2. The choice of postoperative protocol was solely based on the treating surgeons’ preference, without any other influencing factors. This nonrandom allocation may have introduced selection bias and limits the ability to infer causality.

**Table 1.** Exclusion and Inclusion Criteria.

Exclusion Criteria	Inclusion Criteria
Concomitant disabling or painful lower limb diseases before or after BMS (CRPS, fractures)	Symptomatic primary or nonprimary OLT treated with arthroscopic BMS
Concomitant procedures which change the postoperative protocol (ATFL repair, syndesmotic repair, malleolar osteotomy)	Lesion size <15 mm in all 3 dimensions as measured with CT

Abbreviations: ATFL, anterior talofibular ligament; BMS, bone marrow stimulation; CRPS, complex regional pain syndrome; CT, Computed Tomography; OLT, osteochondral lesion of the talus.

**Table 2.** Definitions of Postoperative Rehabilitation Protocols.

Immediate weightbearing	Immediate weightbearing as tolerated from day 1 postoperatively. Crutches needed for the period without full weightbearing if needed 48-h pressure dressing Start ROM exercises after 48 h Start physical therapy after 1 wk
Delayed weightbearing	Nonweightbearing for 6 wk postoperatively Crutches needed for the period without full-weightbearing 48-h pressure dressing Partial to full weightbearing from 6 to 10 wk postoperatively Start ROM exercises after 48 h Start physical therapy after 1 wk

Abbreviation: ROM, range of motion.

### Clinical Evaluation

The following patient characteristics were collected at baseline: age, gender, weight, height, body mass index (BMI), affected side, primary/nonprimary origin of the lesion, and postoperative regimen (ie, immediate weightbearing or delayed weightbearing) as described in Table 2. By means of questionnaires, the following outcomes were collected at baseline and at the 12-month follow-up: numeric rating scale (NRS) scores for the subscales of pain during walking, pain during rest, pain during running, and pain during stair climbing; Foot and Ankle Outcome Score; and 36-Item Short Form Health Survey score. All questionnaires were sent with Electronic Data Capture (EDC) program Castor. Additionally, all patients were evaluated for complications including wound infections within 6 weeks following surgery or reoperations within 12 months after surgery.

### Radiologic evaluation

Radiologic characteristics were collected from the electronic patient files. Measurements were performed by 2 independent researchers on all baseline computed tomography (CT) scans (TB, JH). The following lesion characteristics were measured at baseline and at 12-month follow-up: anteroposterior (AP) size (mm), medial-lateral (ML) size (mm), depth (mm). Based on all diameters, lesion surface (mm^2^) was calculated according to the formula 0.79 × AP × ML, and lesion volume (cm^3^) was calculated according to the ellipsoid formula. The lesion locations were collected according to the 9-grid scale.^7^ Additionally, the volume of bone filling at final follow-up was measured and graded as good (67%-100%), moderate (34%-66%), or poor (0%-33%).^17^ Cyst recurrence was assessed by 2 independent researchers (TB, JH) and was defined as persistence or recurrence of cysts in the treated area.

### Outcome Measures

The primary outcome of this study is the comparison of the change in NRS of pain during walking, between baseline and at 12 months postoperatively between both groups. Furthermore, the proportion of patients exceeding the minimal clinically important difference (MCID) in both groups was calculated. Secondary outcomes consisted of change in the NRS subscale scores of pain during running, pain during stairclimbing, and pain during rest; 36-Item Short Form Health Survey score; Foot and Ankle Outcome Score subscales return to work and return to sport; and radiologic outcomes between both groups at 12 months.

### Return to Sport and Work Outcomes

Preoperatively, data on the level (ie, amateur, competitive, or professional) and type of sports were collected. Return to sport was defined as return to any level of sports. Return to work was defined as return to their own work function with minimal adjustment of working activities.

### Statistical Analysis

Matching in this study was conducted according to the propensity score–matching strategy. The following variables were used for matching: age, gender, side, lesion size (volume and surface), primary or nonprimary lesion, BMI, and pain at baseline measured with the NRS of pain during walking. The sample size calculation was based on a power of 80% with a level of significance of .05. An MCID of 2 with an SD of 1.3 on the primary outcome was used as this correlates with “much better.”^14,19^ This resulted in a minimum of 10 patients per treatment group in order to reach a power of 80%.

All continuous baseline characteristics are presented in mean (SD) or median and interquartile range depending on distribution of the data. Categorical characteristics are presented in absolute numbers and percentages. Normality of the data was determined based on the Shapiro-Wilkinson test and visual inspection.

For the comparison between both groups, an unpaired *t* test or Mann Whitney *U* test was used for the comparison at 12 months in case of numerical data, depending on distribution of the data. In case of ordinal data, a χ^2^ test was used. In all cases, a *P* value less than or equal to .05 was considered as significant. Cohen *d* was calculated to provide a standardized effect size for the difference between both groups on the primary outcome, NRS pain during weightbearing score, when the data were normally distributed, and Hedges *g* was used when the data were not normally distributed.

For the measurement of lesion sizes, an interobserver reliability was calculated using a 2-way random interclass correlation (ICC) model. The ICC was interpreted as poor (0.40), moderate (0.40-0.75), substantial (0.75-0.90), or excellent reliability (>0.90).^22^ Two researchers (TB, JH) assessed the bone filling and cyst recurrence together and consulted a third researcher (JD) in case of doubt or disagreement.

All statistical analysis were performed using R (version 4.1.0; Foundation for Statistical Computing, Vienna, Austria).^16^ Validation of the analyses was performed using custom-made scripts in Python (version 3.11.8) with packages SciPy (1.11.2) and Matplotlib (version 3.7.2).^18,23^

## Results

**Figure 1.**
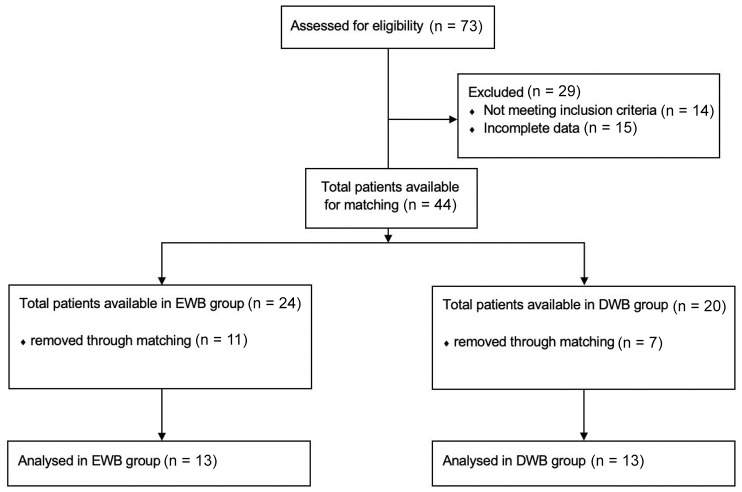
Selection procedure.

In total, 44 patients were eligible for this study. Fifteen patients were excluded because of incomplete baseline or follow-up data of the primary outcome. Fourteen patients were excluded because of additional procedures during surgery. Ten patients received additional ankle ligament surgery, 3 had a syndesmotic repair, and 1 underwent an open BMS. After matching, 13 patients per treatment group were included (Figure 1). Baseline characteristics are presented in Table 3 and the supplementary materials. Twenty patients had a history of ankle surgery, of which 18 patients had previous surgery on their OLT (16 arthroscopic and 2 open). Of these 18 patients, 1 had previously undergone ankle ligament reconstruction and 1 had a flexor hallucis longus release. Three patients had an osteophyte resection.

**Table 3.** Baseline Characteristics.

	Immediate Weightbearing (n = 13)	Delayed Weightbearing (n = 13)	*P* Value
Age, y, mean (SD)	28.4 (10.0)	28.8 (8.3)	.89
Gender, M/F, n	6/7	8/5	.43
BMI, mean (SD)	23.6 (2.7)	23.7 (2.9)	.94
Side, R/L, n	7/6	6/7	.69
Primary/nonprimary, n	2/11	4/9	.18
Arthroscopic approach (anterior), n/n (%)	11/13 (85)	13/13 (100)	.14

Abbreviations: BMI, body mass index; F, female; L, left; M, male; R, right.

### Clinical Outcomes

The mean NRS of pain during weightbearing improved from 5.2 to 3.7 (*P* = .05) points in the immediate weightbearing group and from 5.2 to 3.2 (*P* = .02) points in the delayed weightbearing group. The change in NRS of pain during weightbearing from baseline to 12-month follow-up did not differ significantly between the immediate weightbearing group and the delayed weightbearing group (*P* = .57; 95% CI -3.25-1.86). Sixty-two percent of the patients in the immediate weightbearing group exceeded the MCID of 2, which was 46% in the delayed weightbearing group (OR 1.9, 95% CI 0.30-11.7). None of the other change scores on clinical outcomes on the NRS subscales, as well on the Foot and Ankle Outcome Score subscales, differed between the 2 groups at the 12-month follow-up. A Hedges *g* of −0.25 (95% CI −1.0 to 0.5) is calculated. All clinical outcomes are presented in Table 4. Figures on all other clinical outcomes are presented in the appendix.

**Table 4.** Change in Clinical Outcomes at 12- Month Follow-up.

	Immediate Weightbearing			Delayed Weightbearing			*P* Value Between Groups^a^
	Baseline, Mean (SD)	12 mo, Mean (SD)	*P* Value (95% CI)	Baseline, Mean (SD)	12 mo, Mean (SD)	*P* Value (95% CI)	
NRS during walking	5.2 (2.1)	3.7 (2.7)	0.05 (−0.03 to 2.95)	5.2 (2.6)	3.2 (3.0)	0.02 (0.45 to 3.86)	0.57 (−3.25 to 1.86)
NRS during Rest	3.7 (2.5)	3.2 (2.5)	0.56 (−1.41 to 2.49)	3.8 (1.8)	1.6 (2.1)	0.01 (0.67 to 3.79)	0.15 (−4.88 to 1.50)
NRS during stair climbing	5.8 (2.1)	4.2 (2.8)	0.11 (−0.43 to 3.34)	5.3 (2.6)	4.3 (3.3)	0.08 (−0.27 to 3.54)	0.84 (−3.94 to 3.27)
NRS during running	7.3 (2.2)	5.2 (3.1)	0.004 (0.97 to 4.02)	7 (2.9)	5.4 (3.7)	0.06 (−0.09 to 3.49)	0.55 (−1.52 to 2.63)
FAOS symptoms	48.6 (19.2)	63.5 (15.8)	0.02 (−26.92 to −2.72)	56.0 (21.6)	61.0 (27.0)	0.48 (−19.9 to 10.00)	0.55 (−1.52 to 2.63)
FAOS pain	57.1 (18.5)	70.3 (19.1)	0.09 (−28.72 to 2.18)	52.8 (17.7)	73.5 (20.7)	0.003 (−32.98 to −8.46)	0.49 (−15.33 to 30.23)
FAOS ADL	68.1 (19.0)	79.8 (20.2)	0.10 (−25.95 to 2.57)	66.3 (19.5)	80.4 (18.9)	0.02 (−25.58 to −2.57)	0.75 (−13.72 to 18.49)
FAOS sports	36.9 (20.1)	53.8 (29.0)	0.02 (−31.02 to −2.83)	38.1 (27.9)	55 (24.4)	0.02 (−30.91 to −2.94)	1.00 (−17.27 to 17.27)
FAOS QOL	27.4 (17.0)	42.8 (19.7)	0.03 (−29.16 to −1.69)	26.9 (15.2)	43.3 (21.1)	0.02 (−28.88 to −3.77)	0.91 (−16.37 to 18.18)

Abbreviations: ADL, activities of daily living; FAOS, Foot and Ankle Outcome Score; QOL, quality of life; NRS, numeric rating score.

a *P* value of the change scores between both groups.

No complications or reoperations were recorded in either group. In the immediate weightbearing group, 11 of 11 patients (100%) returned to work after a mean time of 7.3 weeks (SD: 3.7). In the delayed weightbearing group, 8 of 10 patients (80%) returned to work after a mean time of 9.8 weeks (SD: 6.8). Nine of 11 patients (81%) returned to sports after a mean time of 18.8 weeks (SD: 10.3) after immediate weightbearing. After delayed weightbearing, 9 of 10 patients (90%) returned to sport after a mean time of 19.2 weeks (SD: 12.0). Both rate and time of return to sport did not significantly differ between both groups. All return to work and return to sport outcomes are presented in the supplementary materials.

#### Radiologic outcomes

At 12-month follow-up, no significant differences were observed between either group in terms of cyst recurrence or lesion filling. All radiologic outcomes are presented in the supplementary materials. For lesion sizes, an ICC of 0.96 was calculated.

## Discussion

The most important finding of this study is that there is no statistically significant difference in change on NRS pain during weightbearing between baseline and 12-month follow-up between immediate weightbearing and delayed weightbearing. A clinically relevant difference was observed in the proportion of patients who exceeded the MCID of 2, with a higher rate in the early weightbearing group. No other difference in patient reported outcomes, return to sport and return to work, or radiologic differences were found between both groups at the 12-month follow-up. No complications or reoperations occurred within the 1-year follow-up. These results support the feasibility of immediate postoperative weightbearing, but should be interpreted with caution because of the exploratory nature of the study and limited sample size.

Weightbearing after surgical procedures is a point of ongoing debate as early weightbearing provides an early return to normal function and provides better quality of life. However, it has been reported that the fibrin clot created as a result of the BMS needs a certain time of immobilization.^9,21^ On the contrary, another school of thought is that early weightbearing is needed for optimal fibrocartilage maturation. The literature regarding similar cartilage injuries in the knee has shown no adverse effects following early weightbearing, which supports the safety of early weightbearing in cartilage lesions.^10,12^ Additionally, noncompliance has been reported with delayed weightbearing.^21^ These reasons emphasize the need for a study assessing early weightbearing (defined as immediate postoperative weightbearing).

This is the first study that describes clinical and radiologic outcomes after immediate postoperative weightbearing. In the current literature, several definitions have been used for early weightbearing. However, immediate progression to full weightbearing from the first postoperative day without bracing or splinting has not been published before. Wei et al^24^ used braces and progressed to full weightbearing at 4 weeks, whereas the current study does not use any brace and allows full weightbearing immediately postoperation. Early weightbearing and avoidance of supportive plasters or braces enables patients to mobilize early and facilitates early return to normal function. Early return to normal function will improve quality of life and patient satisfaction. Findings of this study showed that there is no difference in clinical outcomes at 12 months while confirming that the benefits of early weightbearing do not negatively influence the outcomes at 12 months.

When compared to studies that start with partial weightbearing and progress to full weightbearing at 4 weeks, it can be stated that there is no difference between immediate postoperative weightbearing in terms of clinical outcomes.^24^ Although no complications or reoperations were observed in this small cohort, the need for bracing should be evaluated cautiously and confirmed in future studies.

This study shows that there were no significant differences at the 12-month follow-up between the immediate weightbearing group and delayed weightbearing group on the primary outcome, NRS during weightbearing. Furthermore, the Hedges *g* of −0.25 could be interpreted as “small difference,” which indicates that there is no relevant difference between both groups on the primary outcome based on the Hedges *g*. Despite the absence of a statistically significant difference, a larger proportion of patients exceeded the MCID in the early weightbearing group compared with the delayed group, raising the possibility that early weightbearing could be a preferable approach, although further research is needed. However, these findings should be interpreted with caution because of the wide CIs, which indicate a high degree of uncertainty. In addition to the higher proportion of patients exceeding the MCID in the early weightbearing group, a trend suggesting an earlier return to sport in the immediate weightbearing group was observed, which can both be observed as clinically relevant. This finding emphasizes the importance of immediate weightbearing in the sporting population that frequently exercises, because one of their most substantial motives might be to return to their sport as quicky as possible. Furthermore, patients who return to function earlier often report better health-related quality of life. These exploratory findings suggest that immediate weightbearing may be feasible, but confirmation in larger studies is needed.^21^

With an early return to sport and high rates of returning to sport in this study, immediate weightbearing should be considered in daily practice. However, results on the return to sport rate need to be interpreted with caution as we do not take into account the level of returning. This should be studied more closely in order to give patients a clear view on what to expect.

Besides the equal clinical outcomes found in this study, even radiologic outcomes were similar as well after 12 months in both groups. Furthermore, there was no difference in complications or reoperations between the 2 groups. This may affirm that immediate weightbearing is safe after arthroscopic BMS in comparison to delayed weightbearing.

### Strengths and Limitations

To our knowledge, this is the first study that describes the comparison between immediate postoperative weightbearing and weightbearing after 6 weeks following arthroscopic BMS. Additionally, to our knowledge, this is the first weightbearing study comparing radiologic outcomes between immediate weightbearing and delayed weightbearing. Another strength is the return to sport and return to work outcomes, which has not been analyzed in literature. Furthermore, use of matching strategy and power calculation can be considered as strengths because this improves the methodologic quality.

However, our power calculation was based on an MCID for the NRS, which is not validated for OLTs. The clinical relevance of this threshold in OLTs remains uncertain. Another limitation is that this study lacks short-term outcomes, which made it difficult to draw conclusions in terms of clinical outcomes and patients’ satisfaction after 3 and 6 months. It is expected that patients would be more satisfied if they could return earlier to normal function, but outcomes of this study do not allow for this conclusion to be drawn as it may be affected by recall bias. Additionally, although patients were matched on key clinical variables, selection bias remains a risk because of the retrospective, surgeon-determined allocation to postoperative protocol. This design limits the ability to control for unmeasured confounding. Furthermore, duration of symptoms and physical therapy treatment before surgery is not included in this study, which might influence the success of the treatment. However, this factor may be affected by recall bias as well. Despite the power calculation, only 13 patients were included in each group. This limited sample size may reduce statistical power, increasing the risk of a type II error and leading to a false negative result. When considering the power calculation for the primary outcome, a large effect size was used based on existing literature. However, this may have contributed to the inability to detect statistically significant group differences within the limited sample size. Future studies need to assess subjective outcomes such as satisfaction and quality of life in the short term to confirm the supposed advantages of immediate weightbearing. Another limitation is the risk of type I errors as a result of multiple testing. In the present study, such a correction would not affect the conclusions drawn from the results, as it was not statistically significant and its exploratory origin. However, future studies with larger sample sizes should consider correcting for multiple comparisons to ensure the robustness of findings.

The current study is conducted in a retrospective manner with a prospective database. Although matching is conducted on the most important factors, a randomized controlled trial with a noninferiority design is needed to assess the outcomes on a higher methodological level. Additionally, the effect of weightbearing on long-term radiologic outcomes is not known.

## Conclusion

This study did not identify any meaningful differences in outcomes between immediate and delayed weightbearing after arthroscopic bone marrow stimulation for talar osteochondral lesions. Because of methodologic limitations and small sample size, the results should be interpreted cautiously and warrant further investigation in larger prospective studies.

## Supplemental Material

sj-docx-2-fai-10.1177_10711007251348196 – Supplemental material for Immediate Postoperative Weightbearing Following Arthroscopic Bone Marrow Stimulation for Talar Osteochondral Lesions Supplemental material, sj-docx-2-fai-10.1177_10711007251348196 for Immediate Postoperative Weightbearing Following Arthroscopic Bone Marrow Stimulation for Talar Osteochondral Lesions by Tristan M. F. Buck, Jari Dahmen, Quinten G. H. Rikken, Julian J. Hollander, Sjoerd A. S. Stufkens and Gino M. M. J. Kerkhoffs in Foot & Ankle International

sj-pdf-1-fai-10.1177_10711007251348196 – Supplemental material for Immediate Postoperative Weightbearing Following Arthroscopic Bone Marrow Stimulation for Talar Osteochondral Lesions Supplemental material, sj-pdf-1-fai-10.1177_10711007251348196 for Immediate Postoperative Weightbearing Following Arthroscopic Bone Marrow Stimulation for Talar Osteochondral Lesions by Tristan M. F. Buck, Jari Dahmen, Quinten G. H. Rikken, Julian J. Hollander, Sjoerd A. S. Stufkens and Gino M. M. J. Kerkhoffs in Foot & Ankle International
