# Supplementary material for: Immediate Postoperative Weightbearing Following Arthroscopic Bone Marrow Stimulation for Talar Osteochondral Lesions: A Matched Cohort Study
Source: Foot Ankle Int. 2025 Jul 10;46(9):943–50. doi: 10.1177/10711007251348196 (PMC12423466; doi:10.1177/10711007251348196)
Supplement: sj-docx-2-fai-10.1177_10711007251348196 – Supplemental material for Immediate Postoperative Weightbearing Following Arthroscopic Bone Marrow Stimulation for Talar Osteochondral Lesions [file sj-docx-2-fai-10.1177_10711007251348196.docx]

# Appendix 1.

Table 1. pre-operative radiological characteristics. n/a = not available, AP=anterior-posterior, ML= medial-lateral, CC= cranial-caudal

|  | Immediate weightbearing (n=13) | Delayed weightbearing (n=13) |
| --- | --- | --- |
| Presence of cysts | 13/13 (100%) | 13/13 (100%) |
| Lesion morphology  Cystic  Fragmentous  Crater | 5/13 (38%)  1/13 (8%)  6/13 (46%) | 5/13 (38%)  0/13 (0%)  7/13 (54%) |
| Lesion Size  AP (mm)  ML (mm)  CC (mm)  Surface (cm^2^)  Volume (cm^3^) | 13.9 (4.3)  8.9 (3.7)  6.8 (2.5)  1.1 (0.9)  0.5 (0.6) | 13.5 (4.6)  9.5 (2.5)  6.5 (2.2)  1.1 (0.5)  0.48 (0.30) |
| Lesion location  1  2  3  4  5  6  7  8  9 | 1/13 (8%)  0/13 (0%)  1/13 (8%)  6/13 (46%)  1/13 (8%)  1/13 (8%)  2/13 (15%)  0/13 (0%)  1/13 (8%) | 1/13 (8%)  0/13 (0%)  2/13 (15%)  4/13 (31%)  2/13 (15%)  2/13 (15%)  2/13 (15%)  0/13 (0%)  0/13 (0%) |

Table 2. Return to sport and return to work outcomes. EWB=Early Weightbearing, DWB=Delayed Weightbearing

|  | Immediate weightbearing | Delayed weightbearing |
| --- | --- | --- |
| Return to Work  Time (weeks). Mean (SD)  Rate | 7.3 (3.7)  11/11 (100%) | 9.6 (8.6)  8/10 (80%) |
| Return to Sport  Time in weeks. Mean (SD)  Rate | 18.8 (10.3)  9/11 (81%) | 19.2 (12.0)  9/10 (90%) |

Table 3. Radiological outcomes

|  | Immediate weightbearing | Delayed weightbearing |
| --- | --- | --- |
| Recurrence of cyst(s) | 3/12 (25%) | 1/12 (8%) |
| Filling  0-33%  33-66%  66-100% | 5/12 (42%)  7/12 (58%)  0/12 (0%) | 5/12 (42%)  4/12 (25%)  2/12 (17%) |
